# Editorial: Women in science: musculoskeletal pain

**DOI:** 10.3389/fpain.2025.1599967

**Published:** 2025-04-28

**Authors:** John Shannonhouse, Yu Shin Kim

**Affiliations:** ^1^ Department of Oral & Maxillofacial Surgery, School of Dentistry, University of Texas Health Science Center, San Antonio, TX, United States; ^2^ Programs in Integrated Biomedical Sciences & Translational Sciences, University of Texas Health Science Center, San Antonio, TX, United States; ^3^ Center for Regenerative Sciences at University of Texas Health and Science Center – San Antonio, San Antonio, Texas, United States

**Keywords:** chronic pain, women in science, pain, musculoskeletal pain, lumbopelvic pain, intervertebral disc (IVD) degeneration, pregnancy-related lumbopelvic pain

**Editorial on the Research Topic**
Women in science: musculoskeletal pain

Sex differences have long been recognized as an important contributor to many aspects of biological phenomena and outcomes but have not been enough studied to the extent of their biological significance merits. Problems that disproportionately or exclusively affect women are widely recognized as under studied (1). Frontiers in Pain Research has devoted a research topic to studying musculoskeletal pain in women. Two areas of musculoskeletal pain that disproportionately affect women highlighted in this topic are lumbopelvic pain (LBPP) and intervertebral disc (IVD) degeneration (Table 1).

**Table 1:** Review of key points and references addressed by this editorial.

Key review subject and paper	Key points
Pregnancy-related LBPP, Daneau et al., 2021	Smaller, mechanistic studies give inconsistent results. Interaction with other kinds of pain. Motor adaptations to pregnancy and their relation to LBPP.
Rodent Models of IVD Degeneration, Tang et al., 2022	Rodents and humans have fundamentally different spinal anatomy. Standardization, translatability, and consistency across models in behavior and markers of IVD may improve the field.

## Pregnancy-related LBPP

Pregnancy-related lumbopelvic pain (LBPP) is a combination of pregnancy related lower back pain and pelvic girdle pain. Study of LBPP on large populations (>70,000) reliably find statistical correlations. However, smaller, mechanistic studies give varying degrees of inconsistent results (Daneau et al., 2). The efficacy of exercises to relieve pain and prevent chronification of pain, and which exercises are appropriate, are controversial (2, 3). Additionally, common problems including the difficulty of translatability of animal models, numerous changes to the body happening simultaneously during pregnancy, and differences and limited applicability of mixed or all-male pain studies contribute to a making a science-based approach to management of pregnancy-related LBPP difficult.

This review by Daneau et al. proposes an integrated approach of studying neuromechanical, physiological (including hormonal), second or later pregnancies, and clinical changes during all trimesters of pregnancy and interactions between these factors to disentangle the mixed evidence of mechanisms of pregnancy-related LBPP. They note one of the strongest predictors of LBPP is prior LBPP, suggesting (along with other evidence) that motor adaptations during pregnancy contribute to later LBPP. In addition to the value of this paper as a review of pregnancy-related LBPP, they argue studies of interactions of risk factors will be fruitful.

Searching PubMed for the most recent (2022 or later) literature on interactions, motor control, and muscles with lumbopelvic pain and pregnancy found several studies about musculoskeletal adaptation during pregnancy and pain. Papers that did not consider pelvic pain or musculoskeletal structure (including exercise interventions) were not considered. There have been attempts to map changes in musculature and lumbopelvic alignment in detail during and after pregnancy (2, 4–6). Interactions with age and disability and muscular strength in areas of the pelvic region are strongly correlated with pelvic pain during pregnancy, but musculature was not predictive (6). However, Palsson et al. found that deep tissue muscle sensitivity during pregnancy was independent of pain (7). There have been limited recently published studies since the Daneau paper to resolve the controversies around exercise as an intervention (8, 9), and it is difficult to separate the effect of exercise from effects of participants disliking exercise (10). Overall, there is not yet enough literature to evaluate the hypothesis of motor adaptations during pregnancy as a major contributor to LBPP. Future studies are needed to conclude the causal relationship and new contributors for LBPP.

## Intervertebral disc (IVD) degeneration

Intervertebral disc (IVD) degeneration is significantly increased after menopause (11) and after hysterectomy (12). There is significant mechanistic evidence from human studies that alterations in estrogen could contribute to IVD (13–15). Dissection of mechanisms and possible interventions may be accelerated by studying rodent models, but, in addition to normal problems correlating human and animal models, there is the obvious problem that humans are large bipeds and rodents are small quadrupeds, which leads to very different IVD stresses.

## Rodent models of IVD

Lower back pain (LBP) is a common disability, and IVD degeneration is a major cause of LBP. In an extensive review with highlights including sex differences, Tang et al. discuss using mouse models to study IVD degeneration. Despite large differences in mouse and human anatomy, they argue mouse IVD morphology, physiology, and behavior is more similar to humans than dissimilar. Aged and “accelerated aging” mouse models are broadly similar to humans including changes in IVD morphology and inflammatory factors. However, intervention efficacy period coincides with mice beginning to die of old age. Mechanical models (IVD puncture, forced bipedal posture, etc.) can be used on younger mice, but work best on lumbar-level IVDs. The review extensively assesses what has been learned from mouse models of IVD. The authors argue standardization in scoring phenotypes could advance assessment of mouse models by making different studies more comparable. The authors identify several common mouse pain assays that are consistent both between mouse studies and with human behavior, discuss how they determine consistency, and discuss why inconsistencies may occur.

The review ends with a discussion of how to assess the best mouse model for a given clinical or mechanistic question about IVD. The authors have contributed an extensive review of mouse IVD models, their advantages and limitations, and make an argument for the most useful aspects of mouse models of IVD.

Rat models have been extensively studied for anatomical and cellular changes but much less in behavior assays. Barbe et al. compared a 2 lumbar intervertebral disc (IVD) puncture (DP-2) and one lumbar IVD puncture (DP-1) to sham surgery female rats as part of an effort to further develop an animal model for study acute to chronic lower back pain (LBP). The paper extensively compares their results to similar papers. They performed a battery of behavior, serum, and post-mortem histological and morphological assays. To the authors' knowledge, this study provides the most extensive set of behavioral testing of this model in the literature.

Searching PubMed for recent (2022 or later) literature on mouse and rat models of IVD degeneration, excluding those which did not study behavior tests, show limited standardization of behavioral tests for mice (16–19) or rats (20–35). More papers used rat models than mouse models. 70% of papers used von Frey on the paws to test mechanical sensitivity, but no other test was used more than 30% of the time (measurements of various behaviors in the open field test). The most translatable and consistent across models behavior tests identified by Tang et al., grip force, tail suspension, and cold allodynia, were assessed in 50, 100, and 75% of mouse papers, respectively. These were rarely assessed for rats, however (21, 7, and 7%). A fourth of the papers described a new model or refinement of an existing model without new interventions. All papers looked at some gross anatomical measurements of IVD. Many looked at molecular or cellular-level anatomical changes (e.g., macrophage polarization, neurite sprouting, etc.), but there was no protein, cellular, or other marker used in a majority of papers. Rodent models of IVD degeneration are far from standardization or consensus on the best experimental approaches.

## Conclusions

Pain disorders disproportionately affect women and girls (1), but mechanistic studies to allow scientifically informed approaches to pain management have continued to be challenging (1, Daneau et al.). Papers outlined above related to this research topic are part of efforts to study sex differences in musculoskeletal pain using epidemiology and more translatable designs in animal models. This will give us a better understanding of sex-specific mechanisms of pain to improve clinical pain outcomes for women worldwide.
